# Low incidence of advanced neurological burden but high incidence of age-related conditions that are dementia risk factors in aging people living with HIV: a data-linkage 10-year follow-up study

**DOI:** 10.1007/s13365-022-01104-0

**Published:** 2022-12-12

**Authors:** Htein Linn Aung, Mark Bloch, Trina Vincent, Limin Mao, Bruce J. Brew, Lucette A. Cysique

**Affiliations:** 1 Departments of Neurology and HIV Medicine, St. Vincent’s Hospital and Peter Duncan Neurosciences Unit, St. Vincent’s Centre for Applied Medical Research, Sydney, Australia; 2 Faculty of Medicine, UNSW, Sydney, Australia; 3 Holdsworth House Medical Practice, Sydney, Australia; 4 Centre for Social Research in Health, UNSW, Sydney, Australia; 5 Faculty of Medicine, University of Notre Dame, Sydney, Australia; 6 School of Psychology, University of New South Wales, Matthews Building, Room 1012, 11 Botany Street, Sydney, NSW 2052 Australia

**Keywords:** HIV, Aging, Incidence, Comorbidities, Neurological disorders

## Abstract

Although increasing research is focusing on age-related comorbidities (ARC) among people living with HIV (PLHIV), no studies have concomitantly assessed non-HIV age-related neurological disorders (e.g., Alzheimer’s dementia). A total of 254 PLHIV and 69 HIV-negative controls completed baseline medical history and cognitive testing. ARC data were collected from medical records over the subsequent 9-10 years and included all types of strokes, all types of dementia, mild cognitive impairment, Parkinson's disease, motor neuron disease (grouped into a non-HIV age-related neurological category), cardiovascular disease, chronic kidney disease, chronic liver disease, chronic lung disease, non-AIDS cancers, osteoporosis, and diabetes. Kaplan–Meier curves assessed differences in the incident rates (per 1000 person year) of groups of ARC as defined above and combined ARC (i.e., development of any of the ARC) among younger (baseline age < 50) and older (baseline age ≥ 50) PLHIV and younger and older controls. Cox-proportional hazard models assessed the individual and interaction effects of HIV status and chronological age, in addition to a range of demographic and clinical variables including historical and baseline HIV brain involvement on the risk of developing combined ARC. Older PLHIV had a higher incidence of cardiovascular disease, osteoporosis, and combined ARC compared to other groups (*p* < 0.05). Incident rate of non-HIV age-related neurological disorders was 2.3 [0.93, 4.79] per 1000 person year. While this incident rate was higher in older PLHIV (5.37 [1.97, 11.92]) than older HIV-negative participants (3.58 [0.18-17.67]), this was not significant. In multivariate analyses, HIV status and chronological age, but not their interaction, and smoking were associated with higher risk of combined ARC (*p* < 0.05). In analyses focusing on PLHIV, older age and taking abacavir/efavirenz/atazanavir/darunavir containing antiretroviral treatments at the time of diagnosis were associated with greater ARC (*p* < 0.05). Non-HIV age-related neurological disorders are uncommon in older PLHIV, where the majority were < 70 years of age at the end of follow-up. However, the greater burden of ARC among older PLHIV, most of which are established dementia risk factors, warrants the establishment of commensurate prevention strategies and greater attention to neurocognitive screening.

**Supplementary Information:**

The online version contains supplementary material available at 10.1007/s13365-022-01104-0.

## Introduction

Despite a considerable decrease in AIDS-related morbidities and deaths, the prevalence of age-related comorbidities (ARC) (e.g., cardiovascular disease (CVD), chronic kidney disease, non-AIDS cancers, and stroke) is rising among people living with HIV (PLHIV) (Brooks et al. 2012; Negredo et al. 2017; Van Epps and Kalayjian 2017). These conditions are seen at a higher rate and at an earlier age among PLHIV compared to HIV-negative persons suggesting premature, accentuated, and accelerated aging (Aung et al. 2019). In the antiretroviral therapy (cART) era, mortality attributable to these chronic ARC may even exceed the AIDS-related mortality among PLHIV (Lau et al. 2007; Palella Jr et al. 2006; Reisler et al. 2003).

Several factors contribute to the increased occurrence of ARC among PLHIV. In addition to lifestyle factors such as smoking and drug and alcohol use, chronic immune activation with associated inflammation and hyper-coagulopathy likely contribute to these conditions (Justice 2010; Wing 2016). Antiretroviral drugs may play a part as well, given there is an increased risk of diabetes and hyperlipidemia among those taking protease inhibitors (e.g., Lopinavir) (Carr et al. 1998; Muya and Kamuhabwa 2019).

Although increasing research is focusing on ARC, most studies were cross-sectional (Goulet et al. 2007; Guaraldi et al. 2011; Negin et al. 2012). In addition, closely comparable controls were not frequently included (Hasse et al. 2011; Moore et al. 2008; Rankgoane-Pono et al. 2018). Moreover, some studies included relatively large numbers of people who are not on cART and/or virally suppressed (Worm et al. 2010; Yang et al. 2021). To understand whether PLHIV despite suppressive cART have higher risks of ARC compared to HIV-negative persons, longitudinal studies with virally suppressed PLHIV and age and lifestyle-comparable HIV-negative controls are typically needed (Aung et al. 2021a).

Critically, none of the previous studies which identified the risk of ARC among PLHIV concomitantly assessed age-related non-HIV neurological disorders (e.g., Alzheimer’s dementia) (Aung et al. 2019). This is important as there is increasing data showing that aging PLHIV may be at increased risk of cognitive decline because of premature, accentuated, and accelerated cognitive and brain aging (Aung et al. 2022; Sheppard et al. 2017; Valcour et al. 2011). Further, no studies have assessed whether historical HIV-related brain involvement and baseline neurocognitive functioning (using actual baseline cognitive scores rather than an estimate such as reading test) including HIV-associated neurocognitive disorder (HAND) have an impact on the development of ARC. Indeed, in lifespan brain health research, there is an increasing understanding that neurocognitive health and overall health have bidirectional relationships (Calderón-Larrañaga et al. 2019). This has been evidenced in NeuroHIV research in the finding that historical HIV-related brain involvement and current mild neurocognitive impairment are *independent* predictors of mortality and functional decline (Cysique et al. 2010; Grant et al. 2014; Rourke et al. 2021; Thaler et al. 2015; Tozzi et al. 2005; Vivithanaporn et al. 2010). However, this research was conducted either before the ARC were more systematically assessed or before the aging of the HIV population became more advanced.

The current study represents a data-linkage extension of a previously conducted study on neurocognition known as the “CNS HAND” study. The data-linkage to determine a range of ARC was possible because the clinic continues to actively care for most of these patients. At baseline (2011–2012), their neurocognitive functioning, mental health and HIV, and general health were assessed. The baseline data have been published (Aung et al. 2021b; Bloch et al. 2016; Kamminga et al. 2017). We observed that premature aging among PLHIV compared to HIV-negative controls was associated with worse neurocognitive functioning (Aung et al. 2021b). Thus, 10 years later, we had the opportunity to determine the burden of ARC including non-HIV age-related neurological disorders.

The overarching aim of the current study was to determine whether PLHIV compared to their age and lifestyle-comparable HIV-negative persons drawn from the same clinic have a higher risk for ARC. ARC data were collected from medical records over the subsequent 9-10 years and included non-HIV age-related neurological disorders (all types of strokes, all types of dementia, mild cognitive impairment, Parkinson's disease, and motor neuron disease), CVD, chronic kidney disease, chronic lover disease, lung disease, non-AIDS cancers, osteoporosis, and diabetes). In addition, we determined the impacts of HIV brain involvement burden and other demographic, lifestyle, clinical, and treatment factors on the overall ARC burden (development of any of the ARC).

## Methods

### Study design

We adopted retrospective cohort study design. The baseline CNS HAND Study was conducted between October 2011 and October 2012 (Aung et al. 2021b; Bloch et al. 2016; Kamminga et al. 2017). Participants were followed up retrospectively until the end of April 2021.

### Study population

The baseline study was conducted at the Holdsworth House Medical Practice in Sydney, a major primary healthcare clinic providing care to PLHIV and those at risk of HIV. A total of 326 participants (254 PLHIV and 72 HIV-negative) who were receiving care at the clinic participated in this study. Exclusion criterion was substance intoxication at the time of assessment. Details of the baseline study have been reported earlier (Bloch et al. 2016; Kamminga et al. 2017). HIV-negative participants were recruited from the same primary care clinic among those who were receiving HIV prevention services at the clinic. During the study follow-up period, three HIV-negative participants seroconverted and, therefore, were excluded from this study analysis.

### Procedures

In the baseline study, participants’ demographics, medical history (e.g., history of hepatitis B and C infections, history of hypertension, and history of psychiatric disorder), and HIV-related medical and laboratory information (e.g., CD4, prior history of AIDS illnesses, and plasma HIV RNA viral load) were collected. In addition, patient mood status was assessed with a 21-item depression, anxiety, stress scale (DASS-21) questionnaire (Lovibond and Lovibond 1995); instrumental activities of daily living (IADL) were evaluated with a modified version of the Lawton and Brody scale (Heaton et al. 2004); and alcohol use disorder (AUD) and substance use disorder (SUD) were measured with the Mini International Neuropsychiatric Interview (Sheehan et al. 1998). Cognitive performance was assessed with a computerized cognitive screening tool known as CogState Computerized Battery (CCB) (Cysique et al. 2006). The procedure to diagnose HIV-associated neurocognitive disorder (HAND) using CCB and IADL assessment data was described previously (Aung et al. 2021b).

In this long-term outcome study, the targeted outcomes (Table 1) were collected by data-linkage from electronic medical records. The ARC (Table 1) include non-HIV age-related neurological disorders, CVD, chronic kidney disease, chronic liver disease, chronic lung disease, osteoporosis, cancers, and diabetes. To identify if PLHIV participants developed HAND over the follow-up, information on incident HAND cases was also collected. Outcome information was searched and collected from patient summary page and clinician notes and letters. To be ascertained of having an outcome condition, the diagnosis had to be noted down in at least two of the clinical notes. Each participant could have more than one outcome during the follow-up period. All the outcome information was collected for each participant. Regarding the date of diagnosis, the earliest known date of diagnosis was recorded. If a participant was lost to follow-up or deceased or was transferred to another clinic during the follow-up period, these dates were also recorded. In the case of death, cause of the death was determined from the medical records. If the cause of death was associated with one of the outcomes of interest and had not been recorded as having this diagnosis in the clinical notes, the participant was also ascertained as an incident case for this particular outcome, and the date of death was assumed as the date of diagnosis. In addition to the outcome data, participants’ current ART status, ART regimen, CD4 and CD8 counts, and HIV RNA at the time of the diagnosis for those who developed any of the outcomes or at the end of follow-up for those who had not developed any of the conditions were also collected from the clinical notes.

**Table 1:** Age-related comorbidities assessed as the long-term outcomes

**Non-HIV age-related neurological disorders** (all types of strokes, Parkinson’s disease, and motor neuron disease, mild cognitive impairment (MCI), and all types of dementia except HIV-associated dementia)
**Cardiovascular disease** (CVD) (atrial fibrillation, ischemic heart disease, hypertensive heart disease, valvular heart disease, and pulmonary hypertension)
**Chronic kidney disease**
**Chronic liver disease** (cirrhosis)
**Chronic lung disease** (chronic obstructive pulmonary disease (COPD))
**Non-AIDS cancers** (all cancers except skin cancers and AIDS cancers)
**Osteoporosis**
**Diabetes**

### Statistical analysis

Incident rates were calculated based on per 1000 person years. To compare the probability of developing the targeted outcomes over time between PLHIV and HIV-negative in the similar age groups, participants were grouped into four groups: younger PLHIV, older PLHIV, younger HIV-negative, and older HIV-negative using 50 years of age (baseline) as the cut-off (< 50, young and ≥ 50, old). Fifty years of age was used as a cut-off because ARC are likely to increase among PLHIV starting from the age of 50 (Blanco et al. 2012). Kaplan–Meier curve was used to illustrate if there is any difference in the probability of long-term outcomes among the four groups. Subjects who had not developed the targeted outcome by the end of follow-up date (31 April 2021) were censored. Lost to follow-up cases (13 PLHIV and 5 HIV-negative), transferred cases (32 PLHIV and 6 HIV-negative), and participants who were deceased not because of any of the targeted outcomes (1 HIV-negative and 8 PLHIV) were also censored. The mean duration of follow-up among participants is 7.25 years (SD = 2.81). The log-rank test was applied to identify the significant differences between groups. For time to event analysis and incident rate calculation, non-HIV age-related neurological disorders were combined into one group; while chronic kidney, chronic lung, and chronic liver diseases were also grouped into one because the numbers of incident cases were low on their own. Participants who had developed an outcome condition before the enrolment into the baseline study were excluded from the analysis for that particular outcome (three for non-HIV age-related neurological disorder, 10 for chronic kidney/liver/lung diseases, 10 for cancers, 16 for CVD, 12 for diabetes, and 11 for osteoporosis).

Multivariate cox regression was used to identify the independent effects of HIV, age (continuous), their interaction, and all the other covariates on the incidence of ARC. For this analysis, all the ARC outcomes were combined into one group to gain adequate power, and this represents overall/combined ARC burden (i.e., development of any of the ARC). Variables to be included in the multivariate model were identified through a univariate analysis. All the variables which had a *p* value < 0.1 were included in the multivariate model.

A separate cox regression analysis was also conducted pertaining only to the PLHIV participants to identify the effects of HIV-related variables. To identify whether historical HIV-related brain involvement (history of central nervous system (CNS) opportunistic infections and HAD before the baseline) and baseline HIV brain involvement history (baseline HAND diagnosis) predicted the risk of ARC among PLHIV, we created a variable termed HIV brain involvement burden (i.e., the combination of both historical and baseline HIV brain involvement) and tested its effect in the regression model pertained to PLHIV.

Next, to further investigate the effect of the HIV brain involvement burden, we conducted sensitivity analyses to evaluate the effects of different historical and baseline neurocognitive function definitions (Nightingale et al. 2014). We tested in separate regression models the individual effects of baseline HAND (yes/no), historical HIV-related brain involvement (yes/no), baseline demographically corrected global cognitive z-score (GZS, overall demographically corrected neurocognitive performance), and baseline age-uncorrected GZS (overall non-demographically corrected neurocognitive performance). In addition, we also tested the interaction effect of historical and baseline HIV brain involvement. Schoenfeld residuals were checked to ensure the variables used in the final regression models meet the proportional hazards assumption. R statistical software was used for all the analyses.

## Results

### Demographic and clinical characteristics in the study sample at baseline (in 2011–2012)

After exclusion of three HIV-negative participants who seroconverted during the follow-up, a total of 323 participants (254 PLHIV and 69 HIV-negative) remained in the study. Table 2 describes the baseline characteristics among participants, and Table 3 outlines HIV-specific characteristics among PLHIV participants. The mean age at baseline among participants was 49.01 (SD = 10.23) (range 20–75). Most of the participants are gay and bisexual men (99%). Fifteen percent of PLHIV participants had a history of AIDS (1993 CDC definition), and 78% had HIV RNA < 50 copies/mL at baseline and 90% at the end of the follow-up. Forty-two percent of PLHIV participants had HAND at the baseline.

**Table 2:** Baseline demographic and clinical characteristics among participants

**Variable**	**Young HIV-negative (36)**	**Old HIV-negative (33)**	**Young PLHIV (138)**	**Old PLHIV (116)**	**All (323)**
***Demographics***					
Baseline age	38.35 (8.12)	58.77 (6.03)	42.56 (5.69)	57.39 (6.35)	49.07 (10.23)^*^
Gender (male)	34 (94%)	33 (100%)	137 (99%)	116 (100%)	285 (99%)^***^
Ethnicity (White)	32 (89%)	30 (91%)	114 (83%)	106 (91%)	282 (87%)
Education (above secondary school)	26 (72%)	27 (82%)	103 (75%)	73 (63%)	229 (71%)
English-speaking background	34 (94%)	33 (100%)	124 (90%)	115 (99%)	306 (95%)^**^
Employed	32 (91%)	20 (67%)	98 (76%)	61 (54%)	211 (69%)^*^
***Comorbidities***					
DASS depression score^a^	8.44 (9.17)	6.12 (1.59)	8.74 (0.78)	7.53 (0.85)	8.01 (9.13)
DASS anxiety score^a^	5.28 (7.65)	4.55 (5.75)	6.84 (7.31)	5.52 (7.21)	5.96 (7.18)
DASS stress score^a^	10.67 (8.47)	9.15 (8.25)	11.3 (9.73)	10.03 (9.14)	10.56 (9.23)
IADL score^b^	− 0.31 (0.62)	− 0.55 (1.18)	− 1.07 (2.51)	− 1.06 (2.76)	− 0.93 (2.38)
AUD	1 (3%)	3 (9%)	17 (12%)	18 (7%)	29 (9%)
SUD	2 (6%)	0 (0%)	39 (28%)	12 (10%)	53 (16%)^*^
Smoking	8 (22%)	5 (15%)	33 (24%)	24 (21%)	70 (21%)
High cholesterol^c^	6 (38%)	11 (46%)	36 (31%)	35 (37%)	88 (35%)
Hypertension	5 (14%)	15 (45%)	20 (14%)	49 (42%)	89 (28%)^*^
History of hepatitis C infection	0 (0%)	0 (0%)	15 (11%)	13 (11%)	28 (9%)^***^
History of hepatitis B infection	1 (3%)	0 (0%)	5 (4%)	15 (13%)	21 (7%)^**^
History of psychiatric disorders	1 (3%)	0 (0%)	5 (4%)	2 (2%)	8 (2%)
History of lifetime depression	11 (31%)	14 (42%)	61 (44%)	44 (38%)	130 (40%)

Data are presented as n (%) for categorical variables and mean (SD) for continuous variables. Chi-square test was used for categorical, and t-test and ANOVA were used for continuous variables to compare participants’ characteristics between four groups

*DASS* depression, anxiety, stress scale, *AUD* alcohol use disorder, *SUD* substance use disorder, *IADL* instrumental activities of daily living

*p =  < 0.001; **p =  < 0.01; ***p =  < 0.05; ****p =  < 0.1

^a^Higher score means higher symptoms

^b^Higher score means less IADL limitations

^c^Cholesterol > 5.5 mmol/L

**Table 3:** HIV disease characteristics among PLHIV participants

**Variable**	**Younger PLHIV (138)**	**Older PLHIV (116)**	**All (254)**
CDC stage C (yes)	19 (14%)	20 (17%)	39 (15%)
Nadir CD4 cell count (cp/mL)	346 (210)	278(169)	315 (195)^**^
Baseline CD4 cell count (cp/mL)	667 (278)	590 (238)	632 (263)^***^
Current CD4 cell count (cp/mL)	772 (324)	666 (279)	724 (308)^**^
Baseline CD8 cell count (cp/mL)	986 (531)	982 (501)	984 (517)
Current CD8 cell count (cp/mL)	899 (446)	910 (512)	904 (476)
Viral Load < 50 cp/mL (baseline)	105 (76%)	93 (80%)	198 (78%)
Viral Load < 50 cp/mL (current)	122 (88%)	104 (91%)	226 (90%)
On ART (baseline)	122 (88%)	111 (96%)	233 (92%)^***^
On ART (current)	134 (97%)	114 (98%)	248 (98%)
Duration of ART at baseline (years)	7.5 (7.2)	12.1 (7.1)	9.61 (7.5)^*^
HIV brain involvement burden (combination of historical HIV-related brain involvement and baseline HAND)	61 (44%)	57 (49%)	118 (46%)
Historical HIV brain involvement^a^	10 (7%)	22(19%)	32 (13%)^**^
Baseline HAND	59 (43%)	48 (41%)	107 (42%)
Age-corrected GZS	− 0.71 (0.54)	− 0.67 (0.62)	− 0.69 (0.58)
Age-uncorrected GZS	− 0.70 (0.55)	− 1.04 (0.66)	− 0.85 (0.63)
**Current ART regimen**^**b**^			
Tenofovir disoproxil fumarate containing regimen	29 (17%)	24 (16%)	53 (16%)
Abacavir containing regimen	29 (17%)	22 (15%)	51 (16%)
Efavirenz containing regimen	2 (1%)	10 (7%)	12 (4%) ^**^
Nevirapine containing regimen	11 (6%)	14 (9%)	25 (8%)
Lopinavir containing regimen	2 (1%)	0 (0%)	2 (1%)
Atazanavir containing regimen	9 (5%)	6 (4%)	15 (5%)
Darunavir containing regimen	14 (8%)	23 (15%)	37 (11%)^***^

Data are presented as n (%) for categorical variables and mean (SD) for continuous variables. Chi-square test was used for categorical, and t-test was used for continuous variables to compare HIV disease characteristics between two groups. All the variables apart from those which are mentioned as current are baseline data

*ART* antiretroviral therapy, *HAND* HIV-associated neurocognitive disorder, *GZS* global z-score

*p =  < 0.001; **p =  < 0.01; ***p =  < 0.05; ****p =  < 0.1

^a^History of CNS opportunistic infections or HIV-associated dementia before the baseline

^b^ART regimen that was being taken around the time of the diagnosis for those who developed any of the outcomes or at the end of follow-up for those who had not developed any of the conditions. Tenofovir, abacavir, efavirenz, nevirapine, lopinavir, atazanavir, and darunavir were particularly chosen to be studied because they are related to one of the outcome conditions based on previous research

### Individual and combined ARC incidence rates in the four age/HIV groups

**Fig. 1:**
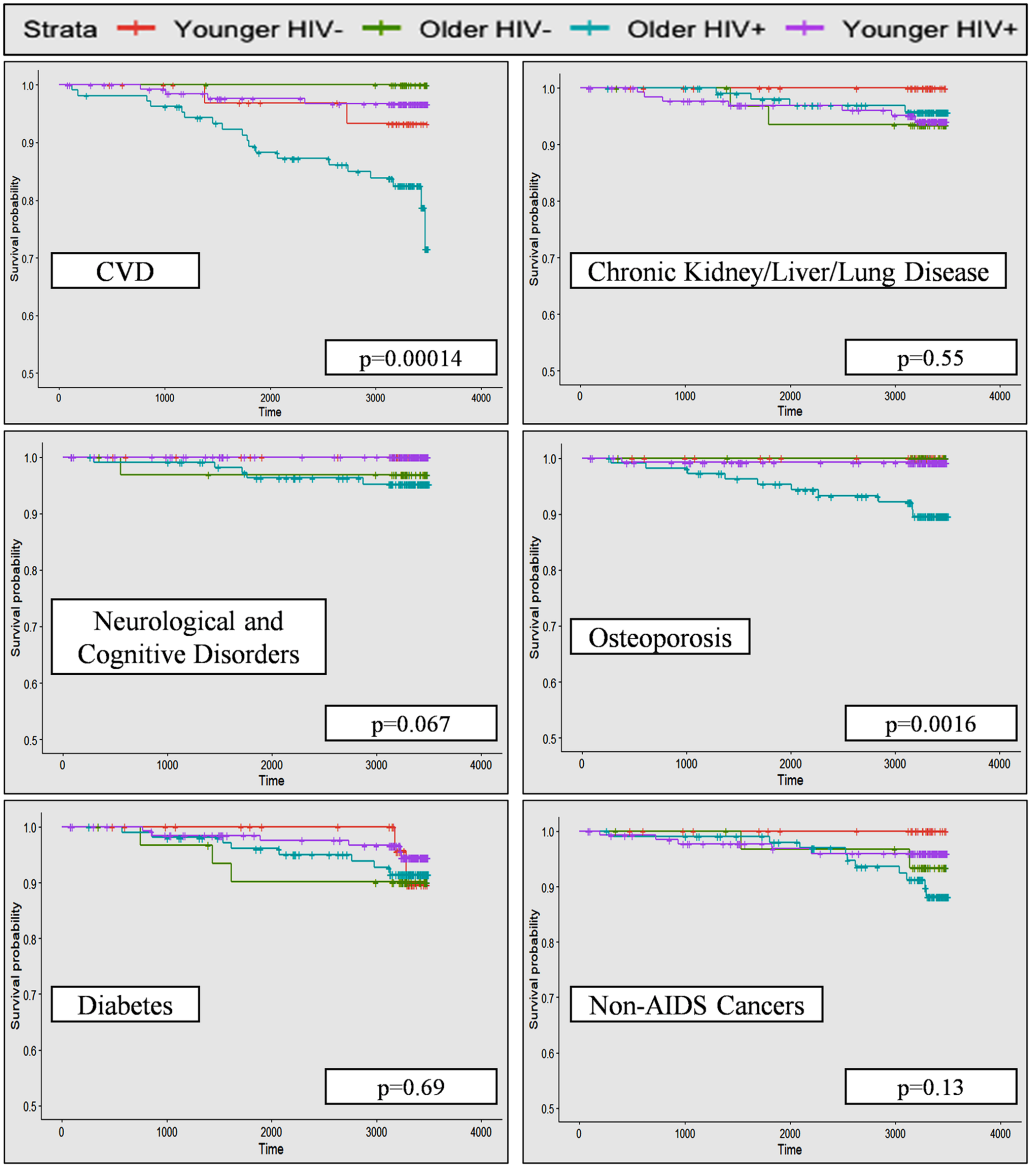
Kaplan–Meier curves of each age-related comorbidity category based on HIV status and age. Time = days. Log-rank test was used to compare the differences in survival probability among four HIV and age groups. Y axis starts from 0.5 because probability does not go down under 0.5 in any of the conditions assessed

**Fig. 2:**
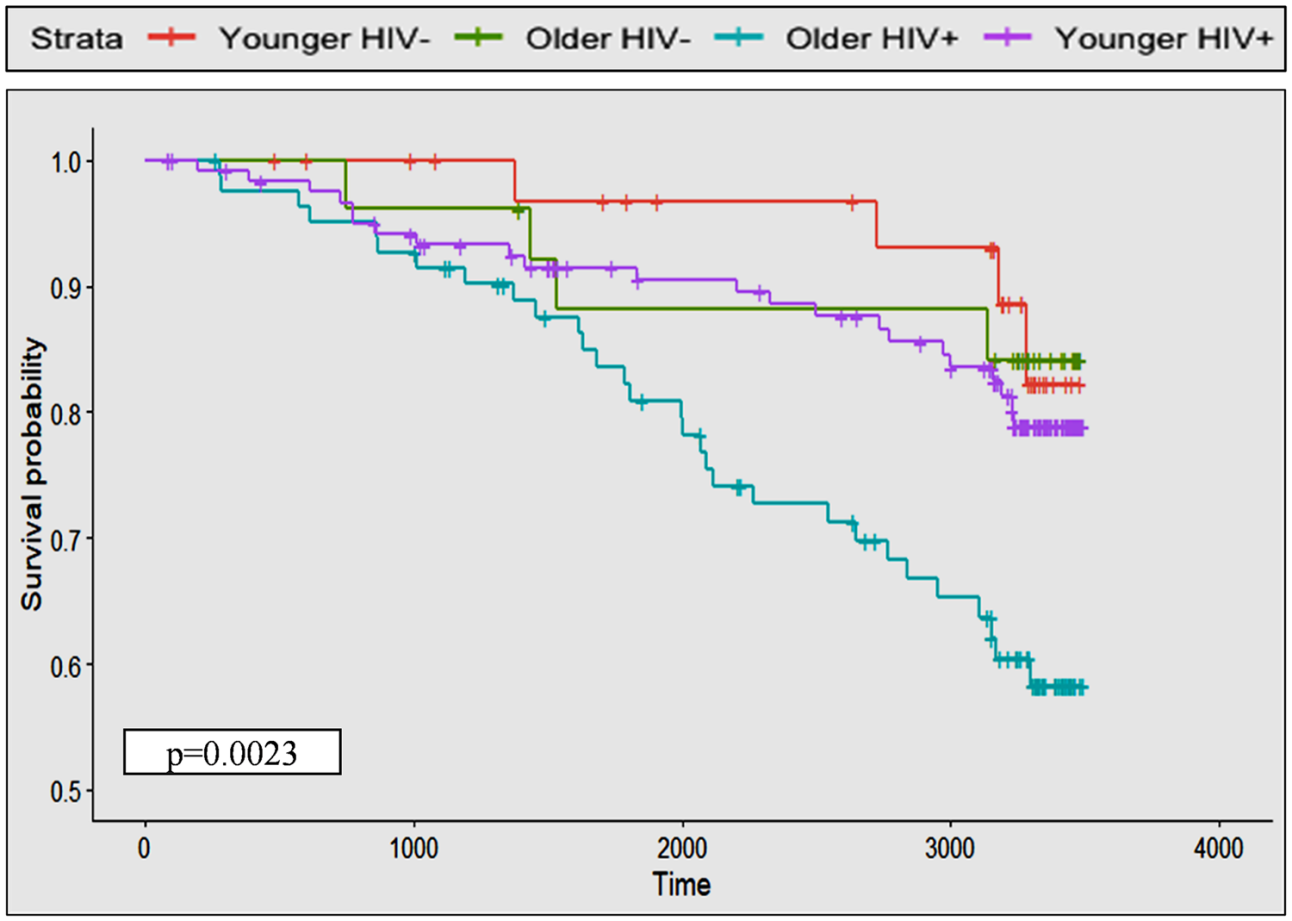
Kaplan–Meier curves for the combined age-related comorbidities by HIV status and age. Time = days. Log-rank test was used to compare the differences in survival probability among four HIV and age groups. Y axis starts from 0.5 because probability does not go down under 0.5

Table 4 presents the incident rate (per 1000 person years) for each chronic ARC category. Over the 9–10 follow-up, six participants developed a non-HIV age-related neurological disorder (one with Alzheimer’s dementia, one with motor neuron disease, and four with stroke), 13 developed a chronic liver/kidney/lung disease, 17 developed a cancer, 25 developed CVD, 19 developed diabetes, and 11 developed osteoporosis (detailed diagnoses under each category were presented in Supplementary File S1). Regarding incidence of HAND, one PLHIV developed asymptomatic neurocognitive impairment (ANI), and two developed mild neurocognitive disorder (MND), but none developed HAD. Overall, the incident rate was the highest for CVD (10.3 per 1000 person years) and lowest for neurological and cognitive disorders (2.3 per 1000 person years) among participants. When the incident rates of ARC groups were compared among four HIV status and age groups, the rate was highest among older PLHIV for non-HIV age-related neurological disorders, cancers, CVD, and osteoporosis, while the incident rates for chronic liver/kidney/lung diseases and diabetes were highest among older HIV-negative participants. The combined ARC incident rate was also highest among older PLHIV participants compared to all other groups. These results are also reflected in Kaplan–Meier survival curves illustrated in Figs. 1 and 2. Log-rank tests showed that the difference in probability of developing ARC among four HIV and age groups was significant for CVD, osteoporosis, and the combined ARC.

**Table 4:** Number of incident cases and incident rate (per 1000 person years) of age-related comorbidities (ARC) among participants

	***Younger HIV-negative***		***Older HIV-negative***		***Younger PLHIV***		***Older PLHIV***		***All samples***	
	**Number of incident cases**	**Incident rate**	**Number of incident cases**	**Incident rate**	**Number of incident cases**	**Incident rate**	**Number of incident cases**	**Incident rate**	**Number of incident cases**	**Incident rate**
***Non-HIV age-related neurological disorders***	0	0	1	3.58 (0.18, 17.67)	0	0	5	5.37 (1.97, 11.92)	6	2.3 (0.93, 4.79)
***Chronic kidney, liver, and lung diseases***	0	0	2	7.21 (1.21, 23.82)	7	6.44 (2.82, 12.75)	4	4.6 (1.46, 11.11)	13	5.16 (2.87, 8.62)
***Cancers***	0	0	2	7.1 (1.19, 23.46)	5	4.6 (1.69, 10.21)	10	11.51 (0.59, 2.05)	17	6.74 (4.06, 10.59)
***Cardiovascular diseases***	2	7.23 (1.21, 23.91)	0	0	4	3.74 (1.19, 9.02)	19	23.08 (14.31, 35.39)	25	10.3 (6.82, 14.98)
***Diabetes***	2	7.06 (1.18, 23.33)	3	11.49 (0.29, 3.13)	6	5.52 (2.24, 11.49)	8	9.27 (4.31, 17.61)	19	7.62 (4.72, 11.68)
***Osteoporosis***	0	0	0	0	1	0.92 (0.46, 45.46)	10	11.3 (0.57, 2.01)	11	4.37 (2.30, 7.61)
***Overall ARC burden (development of any of the ARC)***	4	14.50 (4.61, 34.98)	4	18.54 (5.89, 44.73)	20	21.03 (13.21, 31.91)	29	49.81 (34.00, 70.61)	57	28.15 (21.52, 36.21)

Incident rate was reported for per 1,000 person years with 95% confidence interval

### Predictors of the combined ARC incidence rate in the four age/HIV groups with focus on the age and HIV status effects

Univariate cox-proportional hazard analysis among the total sample (Table 5) showed that HIV status, baseline age, unemployment, higher depression and anxiety symptoms, greater IADL dependence, smoking, hypertension, and a history of psychiatric disorder were associated with a higher risk of developing the combined ARC outcome at *p* value < 0.1. The interaction between HIV status and age was not significantly associated with the combined ARC outcome and, therefore, was not included in the multivariate analysis. In the multivariate model which included variables with *p* value < 0.1 from the univariate analysis, HIV status (HR = 2.23 (1.03–4.82), *p* < 0.05), age (HR = 1.09 (1.06–1.13), *p* < 0.001), and smoking (HR = 2.01 (1.02–3.62), *p* < 0.05) remained significantly associated with a higher risk of developing ARC.

**Table 5:** Cox-proportional hazard model to identify the risk of developing age-related comorbidities among the total sample

	**Univariate**		**Multivariate**	
	**Hazard ratio**	**95% CI**	**Hazard ratio**	**95% CI**
HIV status (positive)	1.98^****^	0.94–4.19	2.23^***^	1.03–4.82
Baseline age (years)	1.07^*^	1.05–1.10	1.09^*^	1.06–1.13
HIV (positive)*** baseline age	1.03	0.96–1.11		
Ethnicity (White)	1.36	0.58–3.16		
Baseline education (above secondary school)	0.64	0.37–1.10		
English speaking background (yes)	1.76	0.43–7.23		
Employed at baseline (yes)^a^	0.39^*^	0.23–0.67		
Baseline DASS depression score	1.03^***^	1.01–1.06	1.02	0.98–1.05
Baseline DASS anxiety score	1.04^***^	1.01–1.07	1.02	0.97–1.08
Baseline DASS stress score	1.01	0.65–2.02		
Baseline IADL score	0.89^**^	0.83–0.96	0.99	0.90–1.09
Current AUD (baseline)	1.07	0.46–2.50		
Current SUD (baseline)	0.58	0.25–1.36		
Smoking at baseline (yes)	1.75^****^	0.99–3.09	2.01^***^	1.12–3.62
Baseline cholesterol level (mmol/L)	1.25	0.96–1.62		
Hypertension at baseline (yes)	1.61^****^	0.92–2.81	1.00	0.54–1.83
History of psychiatric disorder at baseline (yes)	2.97^****^	0.93–9.52	3.37^****^	0.96–11.89
History of hepatitis C infection at baseline (yes)	0.71	0.26–1.97		
History of hepatitis B infection at baseline (yes)	1.18	0.43–3.27		
History of lifetime depression at baseline (yes)	1.14	0.65–2.02		

*DASS* depression, anxiety, stress scale, *AUD* alcohol use disorder, *SUD* substance use disorder, *IADL* instrumental activities of daily living

*p =  < 0.001; **p =  < 0.01; ***p =  < 0.05; ****p =  < 0.1

^a^Baseline employment variable was not included in the multivariate model although it was significant in the univariate model because of missingness among 16 participants

### Predictors of the combined ARC incidence rate among PLHIV and the effects of HIV brain involvement burden

Table 6 shows the results from univariate and multivariate cox-proportional hazard regression analyses which pertain to PLHIV participants only. HIV clinical and treatment variables were added in this model in addition to the demographic and health characteristics. The univariate analysis showed that baseline age, unemployment, higher depression and anxiety symptoms, a history of psychiatric disorders, lower current CD4 count, lower current CD4:CD8 ratio, longer duration on ART, HIV brain involvement burden (combination of historical and baseline HIV brain involvement), current abacavir containing regimen, current efavirenz containing regimen, current atazanavir containing regimen, and current darunavir containing regimen were associated with a higher risk of developing the combined ARC at *p* value < 0.1. In the multivariate model with the variables at *p* value < 0.1 from the univariate analyses, baseline age (HR = 1.07 (1.03–1.12), *p* < 0.001), current abacavir containing regimen (HR = 2.14 (1.12–4.10), *p* < 0.05), current efavirenz containing regimen (HR = 6.42 (2.02–20.31), *p* < 0.01), current atazanavir containing regimen (HR = 3.47 (1.18–10.21), *p* < 0.05), and current darunavir containing regimen (HR = 2.65 (1.27–5.53), *p* < 0.01) were significantly associated with an increased risk for developing ARC. HIV brain involvement burden was not associated with the combined ARC outcome in the multivariate analyses.

**Table 6:** Cox-proportional hazard model to identify the risk of developing age-related comorbidities among PLHIV participants

	**Univariate**		**Multivariate**	
	**Hazard ratio**	**95% CI**	**Hazard ratio**	**95% CI**
Baseline age (years)	1.09^*^	1.05–1.12	1.07^*^	1.03–1.12
Ethnicity (White)	1.19	0.51–2.79		
Baseline education (above secondary school)	0.75	0.42–1.36		
English speaking background (yes)	1.76	0.43–7.25		
Employed at baseline (yes)^b^	0.38^*^	0.22–0.69		
***Comorbidities***				
Baseline DASS depression score	1.03^***^	1.01–1.06	1.03	0.98–1.07
Baseline DASS anxiety score	1.04^***^	1.01–1.08	1.02	0.97–1.08
Baseline DASS stress score	1.01	0.99–1.04		
Current AUD (baseline)	0.95	0.38–2.40		
Current SUD (baseline)	0.52	0.22–1.25		
Smoking at baseline (yes)	1.63	0.88–3.04		
Baseline cholesterol level (mmol/L)	1.29	0.96–1.73		
Hypertension at baseline (yes)	1.19	0.62–2.29		
History of psychiatric disorder at baseline (yes)	3.28^***^	1.02–10.59	3.16^****^	0.87–11.56
History of hepatitis C infection at baseline (yes)	0.61	0.22–1.67		
History of hepatitis B infection at baseline (yes)	1.12	0.40–3.11		
History of lifetime depression at baseline (yes)	1.09	0.59–2.00		
Baseline CDC stage C (yes)	1.61	0.78–3.31		
Nadir CD4 cell count (cp/mL)	0.99	0.99–1.00		
Baseline CD4 cell count (cp/mL)	1.00	0.99–1.00		
Current CD4 cell count (cp/mL)	0.99^***^	0.99–0.99	0.99^****^	0.98–1.00
Baseline CD8 cell count (cp/mL)	1.00	0.99–1.00		
Current CD8 cell count (cp/mL)	1.00	0.99–1.00		
Baseline CD4:CD8 ratio	0.61	0.29–1.25		
Current CD4:CD8 ratio	0.32^**^	0.15–0.66	0.60	0.29–1.24
Viral load < 50 cp/mL (baseline)	1.91	0.76–4.83		
Viral load < 50 cp/mL (current)	0.54	0.34–2.68		
On ART (baseline)	1.95	0.47–08.04		
Duration of ART at baseline (years)	1.07^*^	1.04–1.11	1.03	0.99–1.08
HIV brain involvement burden (combination of historical HIV-related brain involvement and baseline HAND)	1.87^***^	1.06–3.30	1.00	0.52–1.94
**Current ART regimen**^**a**^				
Tenofovir disoproxil fumarate containing regimen	1.94	0.86–4.38		
Abacavir containing regimen	2.13^***^	1.17–3.88	2.14^***^	1.12–4.10
Efavirenz containing regimen	3.61^***^	1.30–10.06	6.42^**^	2.02–20.31
Nevirapine containing regimen	1.12	0.40–3.11		
Atazanavir containing regimen	3.21^***^	1.26–8.12	3.47^***^	1.18–10.21
Darunavir containing regimen	2.54^**^	1.32–4.88	2.65^**^	1.27–5.53

*DASS* depression, anxiety, stress scale, *AUD* alcohol use disorder, *SUD* substance use disorder, *ART* antiretroviral therapy, *HAND* HIV-associated neurocognitive disorder

*p =  < 0.001; **p =  < 0.01; ***p =  < 0.05; ****p =  < 0.1

^a^ART regimen that was being taken around the time of the diagnosis for those who developed any of the outcomes or at the end of follow-up for those who had not developed any of the conditions. Tenofovir, abacavir, efavirenz, nevirapine, lopinavir, atazanavir, and darunavir were particularly chosen to be studied because they are related to one of the outcome conditions based on previous research. Lopinavir was not included in the model because only two participants were taking it

^b^Baseline employment variable was not included in the multivariate model although it was significant in the univariate model because of missingness among 12 participants

Additional sensitivity analyses (Table 7) on the effects of HIV brain involvement burden on the development of the combined ARC among PLHIV showed that historical HIV brain involvement, baseline age-corrected GZS, and age-uncorrected GZS showed significant effects (*p* < 0.05) in the univariate models, but these significant effects were not observed in the multivariate models.

**Table 7:** Additional cox-proportional hazard models to identify whether any of the baseline neurocognitive variables predict the risk of age-related comorbidities among PLHIV participants

		**Univariate**		**Multivariate**	
		**Hazard ratio**	**95% CI**	**Hazard ratio**	**95% CI**
**Sensitivity analysis 1**	**Historical HIV-related brain involvement**^**a**^	2.52^**^	1.06–3.30	1.21	0.52–2.84
**Sensitivity analysis 2**	**HAND diagnosis at baseline**	1.58	0.90–2.78		
**Sensitivity analysis 3**	**Age-corrected global z-score**	0.54^**^	0.34–0.85	0.87	0.50–1.50
**Sensitivity analysis 4**	**Age-uncorrected global z-score**	0.48^*^	0.33–0.68	0.93	0.55–1.57
**Sensitivity analysis 5**	Historical HIV-related brain involvement *** Baseline HAND	0.39	0.09–1.75	1.21	0.52–2.84

*CNS* central nervous system, *HAND* HIV-associated neurocognitive disorder

*p =  <0.001; **p = < 0.01; ***p =  < 0.05

^a^History of CNS opportunistic infections or HIV-associated dementia before the baseline

In multivariate analyses, the following variables were used as covariates: baseline age, baseline DASS depression score, baseline DASS anxiety score, baseline IADL score, history of psychiatric disorder at baseline (yes), current CD4 cell count (cp/mL), current CD4:CD8 ratio, duration of ART at baseline (years), abacavir containing regimen, efavirenz containing regimen, atazanavir containing regimen, and darunavir containing regimen

## Discussion

Our study determined and compared the incidence of common ARC over a 9–10-year follow-up period between clinically stable PLHIV and demographically and lifestyle comparable HIV-negative participants drawn from the same clinic. Unlike previous studies assessing the burden of ARC, we also determined the incidence of non-HIV age-related neurological disorders. In addition, our study is the first to examine the effects of HIV brain involvement burden on the risk of overall ARC burden among PLHIV.

Our findings highlight that PLHIV, despite HIV clinical stability, are likely to develop ARC at a much higher rate than HIV-negative persons as they age. Previous research has also reported the higher prevalence and incidence of ARC among older PLHIV compared to HIV-negative controls (Kong et al. 2019; Lam et al. 2021; Maciel et al. 2018; Onen et al. 2010; Serrão et al. 2019). These comorbidities are established risk factors for dementia in the general population, and their accumulation may further increase the risk of dementia among PLHIV (Cysique and Brew 2019; Montoya 2018; Wright et al. 2010). For effective prevention, management and care of ARC, (Guaraldi and Palella 2017) have proposed that the care for older PLHIV should be led by a multidisciplinary team composed of primary care providers, HIV experts, specialists, and geriatricians. Older PLHIV should be regularly screened for ARC and their risk factors based on the best available evidence and updated guidelines (Martínez-Sanz et al. 2021; Wing 2016). For instance, there should be screening for CVD risk using Framingham risk score (Lloyd-Jones et al. 2004)/D:A:D CVD risk calculator (Friis-Møller et al. 2010) and managing risk factors such as dyslipidemia and hypertension as needed (Lundgren et al. 2008). Polypharmacy is also common among PLHIV along with increasing rates of comorbidities (Halloran et al. 2019; Kong et al. 2019), and possible drug-drug interaction between ART and comorbidities treatment should be considered and managed appropriately (Hughes et al. 2015).

Our study observed a relatively low incidence of non-HIV age-related neurological disorders especially neurodegenerative dementia cases (only one incident case of Alzheimer dementia) compared to other ARC among PLHIV. Although the finding that the incidence of non-HIV age-related neurocognitive disorders is low among older PLHIV despite increasing evidence of premature and accelerated cognitive aging is reassuring, it should be cautiously interpreted. At the end of follow-up, only 9% of our PLHIV participants in this study were ≥ 70 years old, the age at which the prevalence of dementia starts to increase exponentially in the general population (Nichols et al. 2022). Given that dementia risk factors are highly prevalent among PLHIV as reported in our study sample (e.g., high rate of hypercholesterolemia, mid-life hypertension, CVD, and depression), non-HIV dementia risk may significantly increase with aging beyond 70 (Cysique and Brew 2019; Montoya 2018; Wright et al. 2010). Therefore, it is imperative that current PLHIV cohorts are followed until they reach into their 70 s and 80 s to assess the burden of age-related neurological and cognitive outcomes. Another possibility is that the aging effect on neurocognition among PLHIV presents in the form of higher incidence or increased severity of HAND rather than a higher rate of age-related neurodegenerative disorders among older PLHIV (Cohen et al. 2015). In our study, we identified three cases of incident HAND over the follow-up, and all were among older PLHIV. In addition, it is possible that the incidence of age-related neurocognitive disorders especially mild cognitive impairment (MCI) and early dementia is low in our study because of insufficient neurocognitive screening (Löppönen et al. 2003). A systematic review of studies which identified the undetected dementia in community care settings estimated the proportion of undetected dementia at 61.7% ranging between 55 and 66% (Lang et al. 2017). This review also reported that undetected dementia is higher if the age of patients is < 70 and if the patient is cared under a general practitioner (Lang et al. 2017). This may apply to our study, which was conducted in a primary care setting with the majority of participants still < 70 years of age. Because both HIV and age are major risk factors for neurocognitive impairment and as there is increasing evidence of premature and accelerated cognitive aging among PLHIV, regular cognitive screening should be offered to older PLHIV (Chan and Valcour 2022).

In terms of the effect of HIV brain involvement burden on ARC, historical HIV-related brain involvement and baseline neurocognitive function were associated with a higher risk for NCI, but only in the univariate analyses; and there was no independent effect on ARC meaning that we did not detect the effect of brain health on overall health as hypothesized. The significant univariate effects of neurocognitive functioning may in part carry proxy effects of older age, lower education, and higher depression and anxiety symptoms which were significantly associated with historical HIV brain involvement and lower neurocognitive scores at the baseline in our study. Importantly, higher depression and anxiety symptoms have been reported to be strongly associated with ARC in previous research (Hare et al. 2014; Piccirillo et al. 2008; Sartorius 2022). It is possible that this proxy effect of neurocognitive predictors disappeared when the effects of age and depressive and anxiety symptoms were concomitantly tested in the multivariate model.

Our study showed that HIV and age increased the risk of ARC independently rather than synergistically. This finding supports the result from a previous study which analyzed the effect of HIV and age on immune senescence and apoptosis and identified that HIV and age increased T-cell senescence and apoptosis individually rather than synergistically (Hove-Skovsgaard et al. 2020). Another plausible reason for not finding a significant interaction effect between HIV and age on ARC in this study is that our sample size might be underpowered especially within the HIV-negative sample to detect a small-medium interaction effect (Cuzick 1999). Future studies should test the interaction effect of HIV and age on ARC using a larger sample size with at least 50% of the sample representing HIV-negative controls.

Our study supports the finding from previous research that smoking was associated with a higher hazard of ARCs (Brooks et al. 2012; Martínez-Sanz et al. 2021). A Danish study reported that smoking moderates the effect of HIV on myocardial infarction (MI) and association between HIV and MI was only detected among those who had ever smoked (Rasmussen et al. 2015). Managing modifiable risk factors (e.g., stop smoking through smoking cessation programs) may help reducing the burden of ARC among PLHIV (Brooks et al. 2012).

Antiretroviral (ARV) drugs may partly contribute to higher ARC burden among PLHIV (Chawla et al. 2018; Guaraldi et al. 2014). In this study, current treatment with abacavir, efavirenz, atazanavir, or darunavir containing regimen was associated with higher ARC burden. Previous studies have also reported the association between the use of certain ARV drugs and ARC including metabolic disorders (e.g., association between abacavir and cardiovascular events (Choi et al. 2011; Worm et al. 2010); relationship between efavirenz and decrease in vitamin D level and osteopenia (Childs et al. 2012; Dave et al. 2015); increased risk of chronic kidney disease and urolithiasis among those taking atazanavir (Marinescu et al. 2015; Mocroft et al. 2010); and association between darunavir and CVD) (Li et al. 2020; Ryom et al. 2018). Our findings, however, should be cautiously interpreted because the associations between these ARV drugs and ARC burden may not imply a causal relationship since the analysis was based on the current use rather than the cumulative use of these ARVs. Future studies should further explore if there is any causal association between these ARV drugs and the ARC burden using cumulative ART use data. Nevertheless, clinicians should consider individual patients’ factors such as age, lifestyle, and existing medical conditions when selecting ARV drugs and should regularly monitor adverse effects and possible associated conditions (e.g., lipid profile and bone density) (Cardoso et al. 2013).

## Conclusion

This study assessed the incidence of ARC by data-linkage among a group of clinically stable PLHIV and their age and lifestyle matched HIV-negative participants over an average duration of 7.25 years and found that older PLHIV had a higher incident rate of ARC compared to younger PLHIV and younger and older HIV-negative. However, we did not identify a significant interaction effect between HIV and age on ARC although both older age and HIV were significantly associated with higher hazards of ARC showing that age and HIV status in the context of ARC has an additive effect rather than interactive. Non-HIV age-related neurocognitive outcomes had a low incidence potentially because MCI and mild dementia cases were not captured in primary care settings. Nevertheless, evidence that rapid progression towards dementia is not occurring in PLHIV aged between 25 and 75 is reassuring. Clinical and treatment effects were more important than neurocognitive functioning on the overall ARC burden. As the higher burden of ARC among older PLHIV impacts their quality of life, reduces the disability adjusted life years, increases mortality rate, and represents major risk factors for dementia, it is imperative that public health interventions and personalized holistic care plans are needed to prevent, detect early, and manage effectively the ARC among PLHIV as a way to reduce dementia risk.

## Supplementary Information

Below is the link to the electronic supplementary material.

Supplementary file1 (DOCX 15 KB)
