# Supplementary material for: Low incidence of advanced neurological burden but high incidence of age-related conditions that are dementia risk factors in aging people living with HIV: a data-linkage 10-year follow-up study
Source: J Neurovirol. 2022 Dec 12;29(2):141–55. doi: 10.1007/s13365-022-01104-0 (PMC10185650; doi:10.1007/s13365-022-01104-0)
Supplement: Supplementary file 1 — Supplementary file1 (DOCX 15 KB) [file 13365_2022_1104_MOESM1_ESM.docx]

**Table 1: Disaggregate of incident cases under each long-term outcome category**

| Neurological and cognitive outcomes |  |
| --- | --- |
| Alzheimer's disease | 1 |
| Stroke | 4 |
| Motor neuron disease | 1 |
| Chronic Kidney/Liver/Lung Disease |  |
| Cirrhosis of liver | 4 |
| Chronic kidney disease (CKD) | 6 |
| Chronic obstructive pulmonary disease (COPD) | 5 |
| *One participant had both cirrhosis and COPD, and one participant had both COPD and incident CKD.* | |
| CVD |  |
| Ischaemic heart disease | 15 |
| Atrial fibrillation | 4 |
| Hypertensive heart disease | 1 |
| Pulmonary hypertension | 1 |
| Valvular heart disease | 4 |
| Non-AIDS Cancer |  |
| Prostate cancer | 5 |
| Lung cancer | 3 |
| Anal cancer | 2 |
| Renal cell carcinoma | 2 |
| Cholangiocarcinoma | 1 |
| Colorectal carcinoma | 1 |
| Haematology cancer | 1 |
| Rectal cancer | 1 |
| Testicular cancer | 1 |
| Osteoporosis | 11 |
| Diabetes | 19 |
